# The International Medical Annual and Practitioners’ Index

**Published:** 1889-05

**Authors:** 


**The International Medical Annual and Practitioner's Index.** A Work of Reference for Medical Practitioners. By various authors. Seventh year. 8vo, cloth, pp. xx., 544. New York: E. B. Treat & Co. 1889. Price, $2.75.

Although this is the seventh year of publication of the "International Annual," it may serve a convenient purpose to call attention again to its arrangement. The first part is devoted to the subject of *New Remedies*, and this includes not only drugs but new information regarding materia medica in general. The remedies are arranged in alphabetical order, being introduced by a "Review of Therapeutical Progress for the Year 1888," by Percy Wilde, M. D. Under each drug is recorded whatever has been found of value concerning it during the last year. This part also contains two interesting chapters, one on "Mechano-Therapeutics: The Practical Application of Massage in Disease, and the 'Weir-Mitchell' Treatment," by Thos. Stretch Dowse, M. D.; the other on "Electro-Therapeutics," by Kenneth Mullican, B. A., Cantab., M. R. C. S. The second part takes up *New Treatment.* The alphabetical order is followed in this part also, facilitating easy reference. Where a subject would be omitted because no original material had been added during the year, an article by some authority on that subject has been inserted in its place; thus the "Annual" aims to be a complete index for each year. In this issue a new feature appears. Each article is followed by a synopsis of "Remedies and Formulæ," which have been recorded in medical literature during the past seven years, with brief indications for their clinical employment, and references to articles where fuller information can be found. The third part contains a list of books for the year; a list of the principal medical publishing houses; and a list of private asylums and homes for the insane, feeble-minded, and inebriate in the United States.

This is one of the most convenient reference-books that we know. It is practical, concise, and clear. We speak of it, of course, as an *index.* This is what it claims to be, and we think it maintains its claim. Those who desire a complete treatise in any of the subjects introduced will be disappointed, and should look elsewhere, but those who consult it as a work of reference, will find, in great part, what they seek.

The book is well printed, the paper is good, and the type distinct. It is of convenient size, and the price places it within the reach of all.

---

**The Psychic Life of Micro-Organisms.** A Study in Experimental Psychology. By ALFRED BINET. Translated from the French by THOMAS MCCORMACK. 12 mo, pp. xii., 120. Chicago: The Open Court Publishing Company. 1889. Price, 75 cents.

This unique presentation of a subject that has received but little attention, cannot fail to attract the student of microscopy and comparative psychology. The author has grouped in an attractive way such data as are known, and which one must search for elsewhere in a most unsatisfactory manner, scattered and isolated as they are throughout the literature of the lower organisms. The new chapters of the book are replete with scientific interest, and constitute a most fascinating contribution to the study of a subject of growing importance in the field of single-cell life. Some there are, no doubt, who will differ from the author in his views or conclusions, but all must concede that he has taught many lessons of value.

